# Towards a neuro-symbolic approach for precision anti-reflux surgery

**DOI:** 10.1007/s13304-026-02645-3

**Published:** 2026-04-10

**Authors:** Quan Wang, Yaowei Dai, Alberto Aiolfi, Marco Manna, Aldo Ricioppo, Xiaonan Liu, Vincenzo Pezzi, Nicola Leone, Luigi Bonavina

**Affiliations:** 1 Division of General and Foregut Surgery, Department of Pharmacy, Health and Nutrition Sciences, University of Calabria (UNICAL), Azienda Ospedaliera di Cosenza, Cosenza, Italy; 2 Division of General Surgery, Department of Biomedical Sciences for Health, University of Milan, I.R.C.C.S. Ospedale Galeazzi – Sant’Ambrogio, Milan, Italy; 3 Department of Hernia and Abdominal Wall Surgery & Surgical Center for Gastroesophageal Reflux Disease, Xijing Hospital, Ambulatory Surgery Center, The Fourth Military Medical University, Xi’an, China; 4 Artificial Intelligence Laboratory, Department of Mathematics and Computer Science, University of Calabria (UNICAL), Rende, Cosenza, Italy; 5 Department of Pharmacy, Health and Nutritional Sciences, University of Calabria (UNICAL), Rende, Cosenza, Italy

**Keywords:** Gastroesophageal reflux disease, GERD, Anti-reflux surgery, Artificial intelligence (AI), Neuro-symbolic systems, Answer set programming

## Abstract

Surgical management of gastroesophageal reflux disease (GERD) is limited by non-technical challenges, including variability in patient selection, incomplete physiological assessment, imprecise procedure choice, and heterogeneity of intraoperative judgment. Artificial intelligence (AI) offers a promising approach to address these limitations. To enhance decision reproducibility and explainability, we advocate for the integration of AI models based on machine learning with formal logic-based reasoning. This neuro-symbolic approach enables the formal encoding of clinical knowledge, the management of incomplete or conflicting evidence, and the generation of transparent, rule-based recommendations. In the context of antireflux surgery, such hybrid methods are expected to improve decision-making for patient selection and procedure tailoring, and to assist intraoperative quality control for precise hernia repair and optimal wrap configuration. By combining data-driven AI perception with transparent logic, there is the potential to enhance patient’s safety, allow physiology-informed individualized treatment, reduce inter-surgeon variability, and provide better postoperative outcomes.

## Introduction

Gastroesophageal reflux disease (GERD) is a highly prevalent chronic digestive disorder worldwide. It is characterized by the reflux of gastric contents into the esophagus, leading to typical symptoms such as heartburn and regurgitation, along with a range of potential complications. Approximately 5%–15% of patients with GERD may progress to Barrett’s esophagus, a premalignant condition that increases the risk of esophageal adenocarcinoma by 30–50-fold compared with the general population [1].

For individuals with moderate-to-severe GERD, those who fail to respond to proton pump inhibitor (PPI) therapy, or those who cannot tolerate long-term medication, surgical intervention is widely recognized in international guidelines as an effective and potentially curative treatment option [2]. Notably, more than 50% of GERD patients have concomitant hiatal hernia (HH), an anatomical abnormality that exacerbates reflux by compromising lower esophageal sphincter (LES) function, displacing the gastric acid pocket in the mediastinum, and flattening the gastroesophageal flap valve. Therefore, HH repair and cruroplasty is commonly performed in conjunction with fundoplication during antireflux surgery [3].

### Non-technical issues in the surgical management of GERD

Although surgical treatment for GERD has been proven effective, the traditional management pathways and several non-technical clinical bottlenecks continue to limit further improvement in outcomes. These include uncertainty in patient selection, incomplete physiologic characterization, difficulties in selecting the optimal procedure, intraoperative judgment variability, and inconsistency in predicting postoperative functional outcomes [4].

Patient selection for antireflux surgery relies heavily on the surgeon’s clinical judgment, resulting in considerable variability in the assessment of surgical indications and possible adverse events and/or side-effects in a subset of patients [5]. Intraoperatively, the identification of critical anatomical structures—such as the LES region and the crural diaphragm—is often hindered by obesity, liver steatosis or previous abdominal surgery. These factors significantly increase operative complexity and pose particular technical challenges for less-experienced surgeons [6]. Postoperatively, recognition of common symptoms such as dysphagia, chest pain, heartburn, gas-bloating, diarrhea, and dumping remains highly subjective, and many patients are improperly treated and suffer from poor quality of life [7]. Moreover, the absence of objective and quantitative tools for long-term prognostic evaluation makes it difficult to accurately predict persistent symptoms or recurrence risk [8].

Taken together, these limitations not only undermine the overall effectiveness of GERD surgery but also lead to increased healthcare resource utilization. This highlights an urgent need for innovative and intelligent strategies to overcome the current clinical barriers.

## Overview and current evidence of the role of artificial intelligence in anti-reflux surgery

**Fig. 1:**
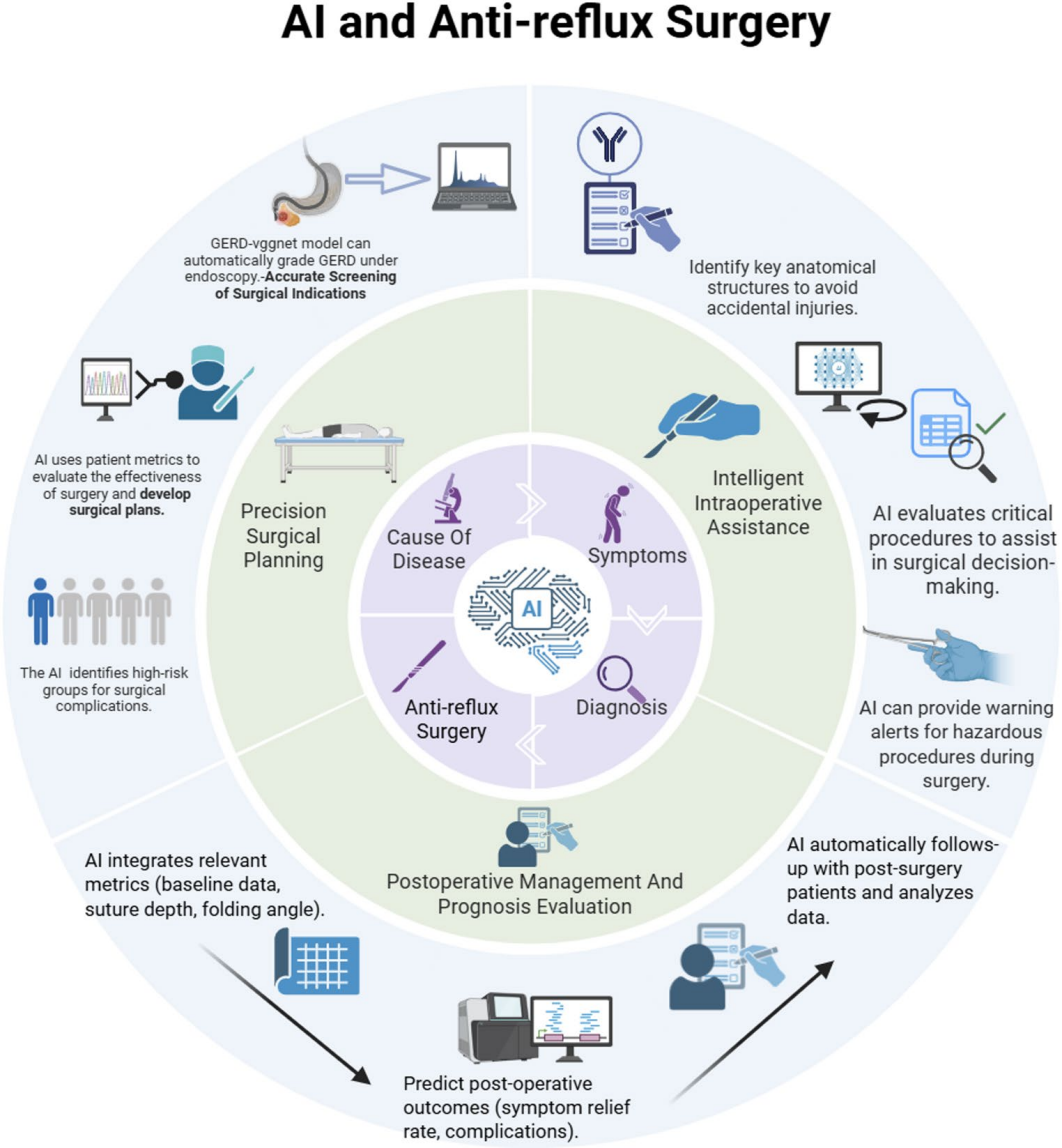
Evidence Mapping of Artificial Intelligence Application Technologies in Anti-Reflux Surgery

The rapid evolution of artificial intelligence (AI) is propelling surgery toward a new era of precision and intelligent care. Current data-driven AI approaches – including machine learning (ML), deep learning (DL), and natural language processing (NLP) – have demonstrated remarkable capabilities in statistical data mining, visual pattern recognition, and information extraction. In the context of GERD surgical management, the strengths of these perceptual models align closely with existing clinical bottlenecks. Preoperatively, ML can integrate multi-source clinical data to support optimized patient selection and surgical planning. Intraoperatively, real-time computer vision enhances the identification of key anatomical structures and strengthens procedural monitoring. Postoperatively, predictive analytics enable early detection of complications and facilitate personalized rehabilitation management (Fig. 1**)**.

### Precision preoperative assessment and optimization of surgical decision-making

#### Indications for surgery

ML models can integrate multi-dimensional data, including clinical symptoms, endoscopic findings, esophageal manometry results, and pH monitoring indicators, to establish a comprehensive assessment system for GERD, thereby accurately identifying patients who are most suitable for surgical intervention. Wang et al. [9] proposed a deep learning model named GERD-VGGNet, which is based on convolutional neural networks (CNNs) to realize automatic classification and interpretation of conventional Los Angeles classification of reflux esophagitis. Experimental data showed that under the narrow-band imaging (NBI) endoscopy mode, the model achieved outstanding classification accuracy in the development set and the independent test set, which was significantly higher than that of trained physicians (75.0% and 65.6% respectively). In addition, the GERD-VGGNet model can realize automatic grading of GERD under both conventional endoscopy and NBI endoscopy. Furthermore, in the context of HH and GERD, ML algorithms can process large-scale datasets derived from endoscopic imaging, radiological studies, high-resolution manometry (HRM), and 24-hr pH-impedance monitoring or 96-hr telepHmetry. This capability facilitates more precise characterization of preoperative anatomical and functional parameters [10,11]. Such comprehensive insights empower surgeons to formulate personalized surgical approaches. By quantitatively analyzing the severity of patients’ condition and the probability of surgical benefits, it can prevent inappropriate long-term medical treatment, reduce unnecessary surgical interventions, and ensure that potential surgical beneficiaries are not missed.

#### Individualized recommendation for surgical approach

Data-driven models can integrate information including patients’ anatomical characteristics, disease severity, and comorbidities, assist surgeons in evaluating the expected efficacy of different surgical procedures, and provide accurate support for formulating individualized surgical plans. In studies on esophageal motility disorders, DL algorithms have analyzed data obtained from high-resolution esophageal manometry (HREM) and functional luminal imaging probe (FLIP), to deeply explore the intrinsic correlation between abnormal esophageal motility and the occurrence and progression of GERD, and to provide objective evidence for logical surgeons’ surgical decision-making based on a pathophysiological perspective [12]. In addition, the size of HH is a core factor determining the occurrence and severity of esophagitis in GERD patients [13]]. With the help of AI technology, standardized evaluation of the gastroesophageal flap valve function has allowed to further clarify the pathogenesis of GERD and its association with the size of HH [14].

#### Preoperative risk prediction

ML models can systematically incorporate diverse data sources, including patients’ clinical baseline information, HREM parameters, endoscopic anatomical features, and comorbidities. These models facilitate the construction of personalized postoperative risk prediction tools, enabling accurate risk assessment of specific complications and surgery-related adverse events. Furthermore, state-of-the-art artificial intelligence algorithms enable automatic 3D reconstruction and segmentation of upper gastrointestinal anatomical structures from preoperative imaging data, allowing surgeons to achieve more precise visualization of complex anatomical correlations and morphological variations. Notably, in complex interventions such as type III/IV paraesophageal HH repair, AI-aided imaging facilitates the accurate quantification of key anatomical parameters—including hernia axial length and volume, esophageal length, and diaphragmatic defect size—thereby improving the precision of surgical planning and performance [15]. Therefore, for high-risk individuals identified by AI models, targeted preoperative intervention plans can be formulated in clinical practice.

### Intelligent intraoperative assistance and precision surgery implementation

#### Anatomical structure recognition and navigation

Through computer vision-based analysis of intraoperative video streams, the integrated application of Computer Vision enables real-time localization of critical anatomical structures [16,17].In minimally invasive surgery for GERD and HH, the narrow surgical field and the limited visual range afforded by 2D laparoscopy can make difficult the real-time identification of key anatomical structures by a surgeon in training. By means of CNN-based systems, key anatomical landmarks relevant to laparoscopic or robotic fundoplication can be automatically detected and labeled, providing surgeons with intuitive visual guidance during the procedure. This capability effectively prevents intraoperative accidental injuries, thereby improving patient’s safety and reducing the incidence of perioperative complications.

#### Auxiliary operation and precision surgery implementation

AI systems can dynamically evaluate and provide real-time feedback on surgeons’ technical steps by collecting and analyzing surgical video streams and surgical instrument movement trajectory data in real time, combined with a preset database of standardized surgical-technical parameters [18]. For GERD procedures, standardization of operative details can directly impact the postoperative recurrence risk [19]. Embedding AI algorithms into robotic surgical systems is transforming the landscape of esophageal surgery by improving instrument maneuverability, mitigating tremor, refining hand motion scaling, and offsetting surgeon fatigue—thereby enabling precise dissection, particularly in the setting of revisional surgery [20]. The potential to standardize surgical outcomes and shorten operative duration not only enhances procedural consistency but also improves clinical efficiency.

#### Intelligent early warning of intraoperative injuries

AI systems can effectively overcome issues such as light and shadow interference in the surgical field, providing higher recognition sensitivity for minimal oozing and occult tissue damage, as well as a shorter early warning response time. Based on accurate identification of critical anatomical structures [21], multi-modal data fusion technology can be applied in the future to achieve accurate capture and real-time response to abnormal signals, to avoid the traditional monitoring method relying on surgeons’ visual observation and empirical judgment.

### AI-Enhanced perioperative management

AI-powered analytical models can process postoperative clinical data, including patient-reported symptoms by wearable technology, to enable the detection of early warning signs prior to clinical deterioration [22]. Clinical studies have confirmed that five core indicators can serve as reliable markers for evaluating the efficacy of antireflux surgery: male gender, body mass index (BMI) < 30 kg/m², presence of typical reflux symptoms, favorable response to proton-pump inhibitor therapy, and abnormal 24-hour esophageal pH monitoring with positive Symptom Index [23]. By integrating and analyzing the above indicators through ML models, precise prediction of efficacy indicators such as postoperative symptom remission rate and reflux recurrence risk can be obtained. This further provides data support for clinicians, not only confirming the necessity of surgery for patients with expected high benefit, but also formulating targeted surgical adjustment plans for patients with expected moderate efficacy [23]. Ultimately, it guides surgeons in formulating more personalized surgical and perioperative management plans.

However, despite its promising potential, current AI applications across the perioperative continuum of anti-reflux surgery remain in an early developmental stage. Most existing studies are exploratory, with notable limitations in quality of evidence, sample size, and dataset scale. First, high-fidelity annotated surgical datasets - which are indispensable for training reliable AI models- are still lacking [24]. Second, even inter-surgeon variability may be recorded in disparate manners and timeframes, eroding label consistency and ultimately compromising model generalizability [25]. Third, AI-assisted anti-reflux surgery introduces novel ethical and legal challenges [26].

Delegating critical decisions based on opaque models entails significant risks. If algorithmic miscalculations cause technical faults leading to recurrence or dysphagia, who holds the primary responsibility—the engineer, the hospital, or the surgeon? Recent work on gradient-weighted class-activation mapping (Grad-CAM) shows that CNNs often base LA-grade classification on vascular pattern rather than mucosal breaks—an observation that would fail regulatory scrutiny [27]. So, to better integrate AI into clinical practice, it’s vital to shift from “black-box” systems to explainable AI, enhancing trust and safety in surgical decisions.

## Logic-based approaches to knowledge representation and reasoning

Logic-based approaches to Knowledge Representation and Reasoning (KRR) provide a formal and interpretable framework for encoding domain knowledge and supporting structured inference. By making expert knowledge explicit through symbolic representations, these approaches enable transparent reasoning over complex decision processes, a crucial requirement in clinical contexts where explainability and traceability are essential. Within this family, Answer Set Programming (ASP) is a declarative logic-based formalism specifically designed for complex reasoning tasks [28–30]. As a highly expressive logic programming paradigm, ASP supports advanced rule sets, non-monotonic reasoning – allowing the explicit handling of exceptions – and optimization constructs. These features make it well suited for modeling intricate clinical decision pathways with mathematical rigor and clarity. Moreover, ASP enables the formal encoding of domain knowledge, supports reasoning under incomplete or potentially conflicting information, and efficiently computes the results, making it particularly appropriate for clinical applications that require explainable, rule-driven inference.

Automated problem solving combined with declarative search-problem specifications has increasingly proven valuable in industry, where ASP provides expressive knowledge-representation capabilities and efficient solvers, enabling robust real-world applications despite challenges such as grounding complexity and runtime optimization [31]. In medical applications, ASP has demonstrated relevant advantages when diagnostic knowledge can be formalized in a structured and symbolic form. A representative example is HEAD-ASP, an ASP-based decision support system designed to infer plausible headache diagnoses from patient-reported symptoms [32]. In this system, the textual diagnostic criteria defined by the ICHD-3 guidelines are manually translated into logical rules, enabling transparent, rule-based reasoning over structured symptom data. Moreover, HEAD-ASP adopts an interactive diagnostic process, posing a minimal and adaptive set of questions to patients or clinicians. Similarly, the full perioperative management pathway for GERD procedures—including preoperative screening and evaluation as well as the procedural steps of antireflux surgery—can be viewed as a “modularized” system of components. So, we cautiously think that Leveraging ASP’s expressive knowledge-representation capabilities and efficient solvers, these modules can be systematically encoded to address the corresponding clinical and logistical challenges.

## Optimal integration points between ASP and clinical practice of anti-reflux surgery

In clinical decision-support contexts, an ASP-based approach can provide a robust framework for reasoning over diagnostic and therapeutic criteria that have been explicitly formalized as logical rules by domain experts, thereby producing consistent, interpretable, and reproducible recommendations.

**Fig. 2:**
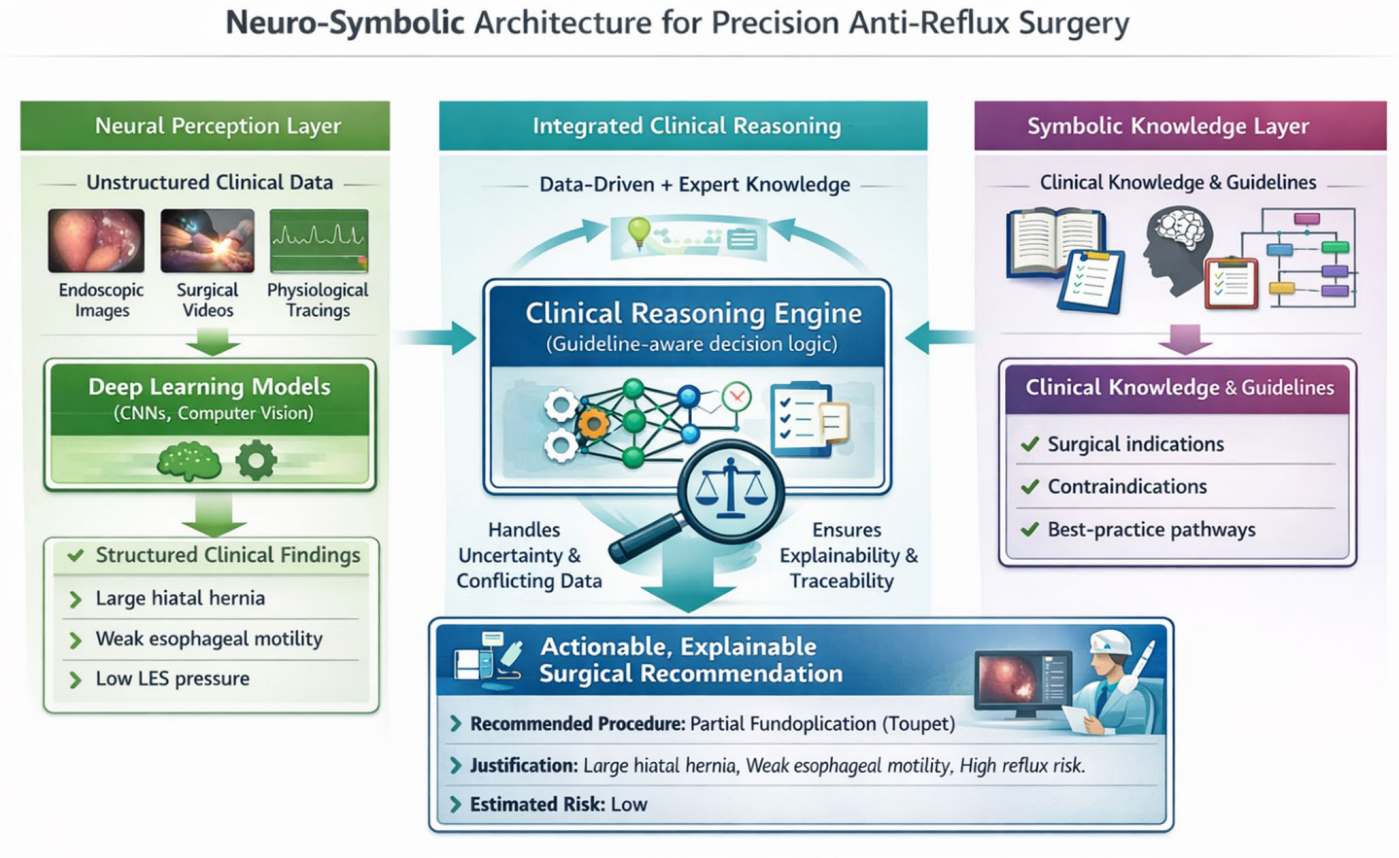
High-level Neuro-symbolic Architecture for Decision Support in Anti-Reflux Surgery. (Multimodal clinical data are interpreted through data-driven perception models and integrated with expert clinical knowledge within an ASP-based reasoning engine, enabling explainable, guideline-aware, and uncertainty-tolerant surgical recommendations)

The above approach allows complex clinical expertise to be structured, formalized, and made computationally traceable, which is essential for decision-making in anti-reflux surgery. It can transform knowledge dispersed across guidelines and expert experience—including GERD phenotypes, pH-impedance metrics, HRM parameters, esophagogastric junction (EGJ) morphology, HH types, anatomical variants of crural diaphragm, esophageal body motility, and the presence of Barrett’s esophagus—into explicit logical rules. This enables the construction of computable clinical logic, the automatic identification of exceptions, and precise “if-then” reasoning, capabilities that cannot be achieved by ML or DL alone. Second, ASP excels in handling incomplete, uncertain, or conflicting clinical information, which is highly characteristic of anti-reflux surgical practice. Common scenarios include incomplete motility data, discordant HRM and pH findings, discrepancies between imaging and symptoms, mismatches between HH size and clinical presentation, and borderline EGJ-D2 physiology. To enhance explainability and decision reproducibility, we advocate for a neuro-symbolic framework that integrates machine learning with formal logic-based reasoning, specifically Answer Set Programming [33–36]. As discussed previously, ASP provides the expressive capabilities needed to manage uncertainty, conflicting evidence, and missing information. By encoding expert domain knowledge into declarative rules, this approach effectively supports the inherent complexity of GERD, HH, and motility-related functional surgery. Figure 2 illustrates the potential applications of this neuro-symbolic integration within the clinical practice of anti-reflux surgery.

### Patient selection

Patient selection remains the main non-technical determinant of success in antireflux surgery, requiring nuanced anatomical and physiological assessment rather than binary indications. Appropriate candidates demonstrate objective reflux and persistent symptoms, whereas those with functional disorders, atypical symptoms, or symptom-reflux dissociation face an high risk of postoperative dissatisfaction. These persistent diagnostic and interpretive uncertainties underscore the lack of structured, transparent, and reproducible reasoning frameworks in the current practice of antireflux surgery, representing an opportunity for neuro-symbolic AI approaches to support standardized, physiology-informed decision-making.

### Procedure selection

Procedure selection in antireflux surgery is a major non-technical challenge requiring the integration of anatomical, physiological, and intraoperative parameters. Rule-based automated decision engines for Answer Set Programming offer transparent and reproducible methods for encoding this complex reasoning. Within such systems, once raw diagnostic findings are encoded as structured logical facts, the rules governing the optimal choice among “complete and partial fundoplication”, “Collis gastroplasty”, “mesh reinforcement”, and “hernia-type-specific repair strategies” can take into account quantifiable parameters such as motility metrics, LES function, EGJ morphology, contractile reserve, and EndoFLIP-derived cross-sectional diameter and distensibility index. By structuring these interdependent conditions into an explainable logic tree, the reasoning engine standardizes decision-making, reduces variability, and enables precise, physiology-informed tailoring of antireflux surgical procedures.

### Intraoperative quality control and surgical decision-making

Intraoperative quality control in antireflux surgery can be substantially enhanced through a neuro-symbolic framework that integrates DL-based computer vision with rule-based symbolic reasoning. DL models can reliably identify key anatomical and procedural features—including diaphragmatic crura, vagus nerve, intra-abdominal esophageal length, extent of mediastinal dissection, tension during crural closure, mobilization of the greater curvature, number of short gastric vessels divided, and wrap geometry (length, width, and tightness). Building on these real-time detections, an ASP-based reasoning engine can evaluate whether each technical step meets predefined quality thresholds, can determine when adjustments are required, and can trigger alerts for unsafe or atypical maneuvers. This combined system forms a neuro-symbolic, real-time, intraoperative quality assurance platform, which offers a structured and explainable approach to model the future of precision surgery.

Moreover, the EGJ barrier in anti-reflux surgery is a multi-component structure involving the LES, the crural diaphragm (CD), the gastroesophageal flap valve and the phrenoesophageal ligament. For example, normal LES pressure with weak CD raises surgical risk; large HH (type III/IV) may require use of a mesh; high EndoFLIP distensibility index (DI) means tighter wrap, and low DI means looser wrap. These physiological measurements interact to define the function of the EGJ barrier as a whole. When integrated into a rule-based logic system, they provide a precise set of decision nodes for tailoring anti-reflux surgery. In other words, the system does not rely on a single parameter but synthesizes multiple components of EGJ physiology to recommend the most appropriate surgical approach in the individual patient.

### Perioperative management

The full perioperative management pathway for GERD surgery—from preoperative screening and physiological evaluation to intraoperative decision-making and postoperative follow-up—lends itself naturally to a modularized computational structure. Using the declarative nature implementation of ASP, each module can be encoded as a transparent set of rules that formalize clinical workflows and reasoning steps. Preoperative tasks can be expressed as declarative logic programs that ensure consistency and reduce interpretive variability. Intraoperatively, ASP-based rules can integrate real-time data from EndoFLIP, manometry, or computer-vision systems to guide procedural tailoring, while postoperative modules can standardize protocols for symptom monitoring, complication alerts, and functional recovery assessment. Through this structured, explainable framework, ASP would offer a scalable method to optimize GERD surgical care, reduce unwarranted variability, and enable reproducible, physiology-informed clinical decision support.

## Current limitations and future perspectives

The field of anti-reflux surgery is constrained by the absence of structured, reproducible, and physiology-centered reasoning frameworks. AI holds substantial promise for improving patient’s assessment and quality of surgery, yet current models remain hindered by limited data availability, high technical inter-surgeon variability, insufficient annotation quality, and the “black-box” nature of many learning algorithms. As the surgical community increasingly demands transparent and accountable AI systems to ensure surgeon’s trust, regulatory compliance, and medico-legal accountability, neuro-symbolic approaches such as ASP frameworks offer a compelling solution by combining data-driven perception with logic-based reasoning.

## Data Availability

All raw data are available if required.
